# Rationale and design of the plate or pin (pop) study for dislocated midshaft clavicular fractures: study protocol for a randomised controlled trial

**DOI:** 10.1186/1745-6215-12-177

**Published:** 2011-07-15

**Authors:** Frans JG Wijdicks, R Marijn Houwert, Marcel GW Dijkgraaf, Diederik H De Lange, Sven AG Meylaerts, Michiel HJ Verhofstad, Egbert JJM Verleisdonk

**Affiliations:** 1 Department of Surgery, Diakonessenhuis, Utrecht, The Netherlands; 2 Clinical Research Unit, Academic Medical Center, Amsterdam, The Netherlands; 3 Department of Surgery, Medical Center Haaglanden, The Hague, The Netherlands; 4 Department of Surgery, St. Elisabeth Hospital, Tilburg, The Netherlands

## Abstract

**Background:**

To describe the rationale and design of a future study comparing results of plate fixation and Elastic Stable Intramedullary Nailing (ESIN) with a Titanium Elastic Nail (TEN) for adults with a dislocated midshaft clavicular fracture.

**Methods/Design:**

Prospective randomized multicenter clinical trial in two level 1 and one level 2 trauma centers. 120 patients between 18 and 65 years of age will be included. They are randomized to either plate fixation or ESIN with a TEN with a one year follow-up. Sixty patients will be treated with plate fixation and 60 patients will be treated with ESIN. Primary outcome parameter is the Disabilities of the Arm, Shoulder and Hand score after 6 months. Secondary outcome parameters are Constant Shoulder Score, complications, experienced pain, radiologic consolidation and cosmetics after both procedures.

**Discussion:**

Prospective randomized studies comparing operative techniques for treatment of dislocated midshaft clavicular fracture are lacking. By studying shoulder function, complications, quality of life, radiographic union, cosmetics as well as experienced pain, a complete efficacy assessment of both procedures will be performed.

**Trial registration:**

The POP study is registered in the Dutch Trial Register (NTR NTR2438).

## Background

Clavicular fractures in adults occur commonly and account for approximately 5% of all fractures. Around 80% of the clavicular fractures involve the midshaft and over half of these fractures are dislocated [1,2]. Historically, clavicular fractures were treated conservatively, mostly with sling or figure-of-eight bandage [3,4]. Consolidation was achieved within a few weeks, even with severe dislocation.

Recently, poor results were described of conservatively treated dislocated midshaft clavicular fractures (DMCF) [5-10]. The number of nonunions after conservative treatment appeared to be much higher than previously assumed [5]. Furthermore, the clinical importance of clavicular malunion was discovered with symptoms like persistent pain, permanent loss of strength, rapid fatigability of the shoulder joint and disappointing cosmetic results [6-8]. Altogether these symptoms result in decreased patient satisfaction scores after conservatively treated DMCF [9,10].

Two commonly used operative techniques for treatment of DMCF are plate fixation and Elastic Stable Intramedullary Nailing (ESIN) [11]. In recently reported prospective randomized studies, functional results after both techniques proved to be superior compared to conservative treatment of DMCF [12,13]. Furthermore, a recent meta-analysis demonstrated a significantly lower nonunion rate after surgical treatment in general [8].

Prospective randomized studies comparing operative techniques for treatment of DMCF are lacking [14]. The aim of this article is to describe the rationale and design of a prospective randomized study comparing results of plate fixation and ESIN with a Titanium Elastic Nail (TEN).

## Methods and Design

### Study design

**Figure 1:**
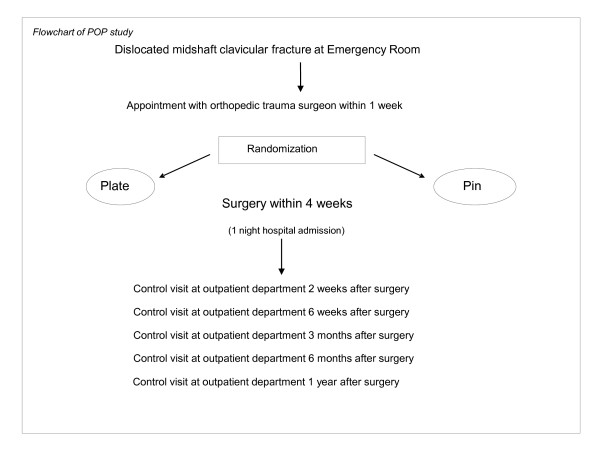
**Flowchart of POP-study**.

Prospective randomized multicenter study involving three hospitals in The Netherlands, including Diakonessenhuis, Utrecht (level 2 traumacenter); Medisch Centrum Haaglanden, The Hague and St. Elisabeth Hospital, Tilburg (both level 1 traumacenters). Patients with DMCF, defined as at least one shaft width difference in height between the fracture parts regardless of the reduction, are allocated to either plate fixation or intramedullary fixation with ESIN through randomization. A flow chart of the study is shown in Figure 1.

### Patient population

A total number of 120 patients will be included in the trial (see sample size considerations).

Patients will be recruited at the emergency room (ER) of the participating hospitals. Patients are screened for eligibility according to the criteria listed in table 1. Patients with a DMCF on one side and no contraindications for general anesthesia are eligible for inclusion in the study.

**Table 1:** Inclusion and exclusion criteria

Inclusion criteria	Exclusion criteria
-Unilateral dislocated midshaft clavicular fracture	-Age < 18 years or > 65 years
-No medical contraindications to general anaesthesia	-Multitrauma patients
-Provided informed consent	-Open fracture
	-Pathological fracture
	-Fracture > 1 month old
	-Neurovascular disorders
	-Glasgow Coma Scale <12 (moderate to severe head injury)
	-Inability to comply with follow-up (for example due to an inability to read or complete forms)
	-Pre-existing shoulder pathology in affected side (rotator cuff lesion, acromioclavicular pathology or previous shoulder surgery)

### Intake

Ultimately within 1 week, an appointment with an orthopedic trauma surgeon and the investigator will take place. If informed consent is obtained, the patient is included in the study, patient data are obtained by the investigator (table 2) and the patient is randomized.

**Table 2:** Preoperative data

Preoperative data	
- Age	- Smoking
- Gender	- Medical history
- Dominant arm	- Medication
- Trauma mechanism (sports, traffic accident, etc)	- SF-36 Questionnaire [18]
- Sports (if yes; at what level? Recreational or professional)	- Body Mass Index
- Occupation	- AO Classification of fracture *

* this classification exactly concurs to the OTA classification of midshaft clavicular fractures

### Randomization

Patients are randomized prior to surgery in the doctor's office by computerized block randomization for either plate fixation or ESIN. The block varies between 2, 4, 6 and 8 patients. In each block the two operative techniques are equally represented. This randomization procedure will be stratified by participating hospital. After randomization, follow-up of patients will take place according to the intention-to-treat principle.

### Interventions

After randomization the patient will be scheduled for surgery as soon as possible but ultimately 4 weeks after the initial trauma.

#### Operative procedure plate fixation

Patients are administered prophylactic antibiotics. With general anaesthesia, the patient is positioned in a beach-chair semi-sitting position. The involved shoulder is prepared and draped, and an incision is made just under the fracture site. If possible, supra-clavicular nerves are identified and spared. The fracture site is identified. In simple fractures, the fracture is reduced and a small fragment, low contact compression plate is fixed upon the anterosuperior surface of the bone starting medially using bicortical, non-angular stable screws. On the outer sides of the plate angular-stable screws are placed. In oblique or complex fractures interfragmentary lag screws can be placed to obtain compression. In case of severe comminution only bridging plate is performed. The fascia and the skin are closed in layers.

#### Operative procedure ESIN using a TEN

Patients are administered prophylactic antibiotics. With a general anesthetic, the patient is placed in supine position. A small skin incision is made approximately 1 centimeter lateral to the sternoclavicular joint. The anterior cortex is opened with a sharp pointed reamer while care is being taken to not accidentally perforate the thorax. A TEN is inserted (the diameter varies from 2 to 3,5 mm, dependent on the width of the bone). Closed reduction, eventually supplemented with two percutaneously introduced pointed reduction clamps, is performed under fluoroscopic control. If closed reduction fails, an additional incision will be made above the fracture site for direct manipulation of the main fragments. After complete introduction of the TEN in the lateral fragment, the fracture is compressed and the TEN will be cut as short as possible at the medial end. The fascia and the skin are closed in layers.

### Postoperative management

If possible, surgery is performed as a day case. Postoperatively, patients are given a sling but are encouraged to start with pain-dependent mobilisation immediately and to discard the sling as soon as possible. Load bearing is not recommended before osseous consolidation. Patients are advised to take pain medication when necessary. The type and amount of analgesics should be kept in the pain diary.

### Follow-up

The patient is requested to record - on a daily basis during the fortnight immediately following surgery - the pain experienced as well as the type and amount of analgesics used. Experienced pain is assessed with a 10-point Likert scale (0 = no pain and 10 = extremely painful).

All patients are reviewed in the outpatient department by the treating surgeon and investigator at 2 and 6 weeks, 3 and 6 months and 1 year after surgery. All visits include standardized clinical evaluation and registration of possible complications by the treating surgeon and the investigator (table 3).

**Table 3:** Complications

Intra-operative complications	Post-operative complications
- Nerve/vessel damage	- Wound healing disorders (infection, hypertrophic scar, dehiscence)
- Other operative complications	- Transient brachial plexus laesion (defined as paresthesia of the arm, and weakness of the pink and index finger)
	- Irritation of the implant (post-operative pain/itch/redness/irritation)
	- Migration of the implant
	- Breakage of the implant
	- Non-union (defined as lack of radiographic healing with clinical evidence of pain and motion at the fracture site after 6 months)
	- Mal-union (defined as union of the fracture in a shortened, angulated, or displaced position with weakness, easy fatigability, pain with overhead activity, neurologic symptoms, and shoulder asymmetry)
	- Other complications

At the 2 weeks outpatient visit the pain diary is discussed by the researcher with the patient. Radiographs will be taken in order to check implant position and, at subsequent follow-up visits until radiographic union. Radiographic union is defined as complete cortical bridging between proximal and distal fragments on both radiographs as determined by the treating surgeon.

The Disabilities of the Arm, Shoulder and Hand (DASH) and Constant scores will be gathered at the 6 weeks, 3 and 6 months and 1 year postoperative visits by the investigator [15-17].

The DASH questionnaire is a self administered region-specific outcome instrument developed as a measure of self-rated upper extremity disability and symptoms. The DASH questionnaire consists mainly of a 30-item disability/symptom scale, scored 0 (no disability) to 100 (= completely disabled upper extremity) [15,16]. To prevent bias, the DASH questionnaires will be completed in absence of the operating surgeon, before the clinical assessment.

The Constant score includes an analysis of pain, shoulder motion, strength, and function. From a perfect score of 100, it reserves 35 points for patient-reported subjective assessment, including the presence of pain and the ability to perform basic activities of daily living, and 65 points for objective measurement. For the latter, 40 points are allocated to range of motion and 25 points are allocated to strength [17].

The cosmetic result after 6 weeks, 3 and 6 months, and 1 year is assessed by eliciting a patient satisfaction score on a 0 (= very unsatisfactory) to 10 (= very satisfactory) Likert scale.

After 6 months and 1 year the patient is asked to complete the SF 36 questionnaire again (see table 2) [18]. The SF 36 is a validated questionnaire designed to measure health related quality of life.

### End Points

The primary endpoint is the DASH score 6 months after surgery. Secondary endpoints are listed in table 4.

**Table 4:** Primary and secondary endpoints

Primary Endpoint	Secondary Endpoints
- DASH score after 6 months	- Constant Score after 6 months
	- DASH and Constant Score after 6 weeks, 3 months and 1 year
	- Complications: intra-operative, post-operative period (2 weeks) and after 6 weeks, 3 and 6 months and 1 year
	- Reoperation after unsatisfying result in a time horizon of 1 year (including implant removal)
	- Time to radiological consolidation, with a maximum time horizon of 6 months
	- Pain score, until 2 weeks postoperative
	- Cosmetic satisfaction after 6 months and 1 year

### Implant removal

Implant removal is scored as a re-operation if it occurs within the first 6 months and is due to implant related problems (listed in table 3).

Implant removal according to the patient's wish will be granted after consolidation.

### Safety measures

Surgeons operating patients for this study must have extensive experience with both plate fixation and ESIN with a TEN. It is assumed that every surgeon must have performed over 20 procedures in both techniques to operate on patients who participate in this study. The cut-off value of 20 operations is established on personal experience by one of the authors (MHJV), who participated in the Content-study in which conservative treatment of DMCF was compared to ESIN with a TEN (results not published yet). In this study the learning curve for ESIN with a TEN was passed after 20 procedures.

### Sample Size and Power

The primary outcome measure in this study is the DASH score. Initially, a minimum difference of 10 points in DASH score can be considered as clinically relevant [16]. However the DASH score rates the whole upper extremity and a smaller difference should be considered as clinically relevant when focusing on clavicular function in particular.

Unfortunately, no studies comparing plate fixation and ESIN for dislocated midshaft clavicular fractures with power calculation are available [14]. Therefore we performed a power calculation based on the following rationale. A DASH score of a "normal" upper extremity varies between four and eight [15]. The DASH score of the group of patients who were conservatively treated for DMCF after 24 weeks was 14 [12]. We consider the difference of six points between this latter DASH score and the worst score (eight) within the normal range as the clinically relevant margin. This coincides well with a recently online published protocol for treatment of wrist fractures in which a comparable margin in DASH score for local function (five points) is considered clinically relevant (Design Minimax studie: http://www.cruamc.nl/Minimax). With an expected standard deviation of 11 points in the DASH score, a two sided alpha of 0.05 and a power of 0.80, 53 patients in each group are needed (total 106) to show a difference of at least 6 points in DASH score after 6 months. Considering that an interim analysis is planned (see below), it is assumed that 2 sequential tests are made using the O'Brien-Fleming spending function to determine the test boundaries. Further assuming a 10% loss to follow-up, 120 patients should be included.

### Statistical Methods

Data will be analyzed according to the intention-to-treat principle. The difference between the operative techniques at the end of the follow-up period in DASH (primary outcome) and Constant scores will be tested for significance using the Student's T-test or the Mann-Whitney U test, depending data distributions. A general linear random effects model will be applied to assess differences during the follow-up period in DASH, Constant, pain, and SF-36 scores to account for repeated measures within patients. The difference in the frequency of complications during follow-up will be assessed with Poisson regression, whereas the Chi-square test or Fisher's exact test will be used to test for differences in proportion of re-operated patients. Differences in cosmetic satisfaction will be tested using the Mann-Whitney U-test. Time to radiological consolidation will be assessed by Kaplan-Meier analysis. A p-value of < 0.05 will be considered statistically significant in all analyses. SPSS software will be used for statistical analysis.

### Data and safety monitoring board and interim analysis

A data and safety monitoring board (DSMB) is established existing of two surgeons and one methodologist. The DSMB will perform an interim analysis after one year. In the interim analysis discrepancies in a) major complications, b) minor complications and c) DASH scores between both procedures are calculated.

Major complications are defined as intra-operative nerve or vessel damage resulting in prolonged hospital admission, persistent injury or death, and re-operation due to an unsatisfying result. Minor complications are the other complications listed in table 3.

After one year, 72 patients (36 patients in each arm) will be included if inclusion is moving on as expected. The following stop criteria are defined:

* an established difference in patients with major complications of 13.5% (1% in one arm against 14.5% in the other) with a two-sided p-value of 0.1, 80% power, and assuming a negligible mortality rate, or

* an established difference in patients with minor complications of 37.1% (15.5% in one arm against 52.6% in the other arm) with a two-sided p-value of 0.01 and 80% power, or

* an established difference in DASH-score with an effect size of 0.55 with a two-sided p-value of 0.003 and 16.5% power (reflecting the first of the 2 sequential tests for the primary outcome using the O'Brien-Fleming spending function), or

* a potential difference in mortality, at the discretion of the DSMB.

### Current status

This study has been approved by the Medical Ethics Committee of the Diakonessenhuis Utrecht. Approval of the local Ethical Boards of the other two participating hospitals is currently requested. This study is performed in accordance with the ethical standards of the Declaration of Helsinki. Recruitment of patients started in January 2011 in the Diakonessenhuis, Utrecht and will start in April 2011 in the Medisch Centrum Haaglanden, The Hague and St. Elisabeth Hospital, Tilburg. To date 25 patients have been included in the study. After a start-up phase the speed of the inclusion is expected to increase steadily and, depending on the number of patients needed to be included in the trial (see sample size considerations), recruitment of the 120^th ^patient is currently expected in July 2012. Analysis and reporting is subsequently expected one year later to be complete (July 2013). The POP study is registered in the Dutch Trial Register (NTR 2438).

## Discussion

No comparative prospective or randomized study has been published comparing outcome of plate fixation with ESIN with a TEN of DMCF. The study aim is to provide and compare results of plate fixation and ESIN with a TEN.

Traditionally DMCF were treated conservatively. This policy was based on good results from large cohort series from the sixties: non union rates were < 1% [3,4].

These series, however, were all very mixed with regard to age, clavicular fracture site, displacement and fracture classification. Children, who have better bone healing and remodelling mechanisms, were also included. Malunion was not yet accepted as a clinical entity. Outcome was surgeon based contrary to present-day patient based outcome tools like DASH and Constant scores.

Surgical treatment however, has its own drawbacks. Wound healing disorders, infections, loss of fixation and nonunions do also occur as listed in table 3[12,13,19-22]. In addition, a second surgical procedure might be required to remove the implant. Nevertheless, recently published studies reporting lower nonunion rates, improved functional outcome, faster mobilization and (perhaps therefore) increased patient satisfaction initiated a tendency towards surgical treatment of DMCF [8,12,13,22].

Theoretically, both plate fixation and ESIN have their own advantages. A biomechanical study shows that plate fixation provides a more rigid stabilization compared to ESIN. Therefore plate fixation may provide a stronger construct for early rehabilitation protocols [23]. Plate fixation is technically easy to perform which provides another advantage.

On the other hand, ESIN is less invasive, results in lesser implant prominence and implant removal can be done with minimal dissection [22]. If closed reduction is possible, this technique has the advantage of an intact fracture hematoma, which could speed up the healing process. However, minimally invasive techniques exert certain specific risks that can lead to complications (e.g. for the clavicle iatrogenic brachial plexus injury have been described [20]). The primary endpoint of this study is the DASH score 6 months after surgery. After 6 months the nonunion rates can be calculated and therefore 6 months is the first possible endpoint to determine the success rate of the surgery. Patients will be followed for a 1 year period, mainly to asses the follow-up of nonunions or other implant-related complications.

The main goal of this study is to compare two principles of osteosynthesis: plate fixation and ESIN. Therefore the operating surgeon is free in his choice of plate regarding compression or angular stable locking. The operating surgeon is also free to decide the location of the plate, both superior and anterior/inferior plating are allowed. If plate fixation proves to be superior, a following study should be initiated to compare different methods of plate fixation.

There are some limitations of this study. Due to different incisions blinding is not possible. However, by using a self administered outcome instrument, the investigator-related bias is minimised for the primary endpoint. In this study the DASH score is used as primary endpoint. The DASH score does not specifically focus on clavicular function. However, a score which solely assesses clavicular function is lacking. In our opinion the DASH score provides the most reliable result for rating upper extremity disability and symptoms. To provide a complete overview of shoulder function the Constant score is used as a secondary endpoint.

A limitation of multicenter studies in general is that patient follow-up is often performed by multiple doctors resulting in decreased consistency of the clinical evaluation (interobserver bias) and increased loss to follow-up. In this study the same investigator (FJW) will be present during all patient visits at the outpatient department. Therefore, the consistency of the results will be improved and loss to follow-up rates should be reduced.

This prospective randomised multicenter study is designed to compare plate fixation and ESIN with a TEN for DMCF. As shoulder function, complications, quality of life, radiographic union, cosmetics and experienced pain are assessed, this study will provide a complete efficacy assessment of both procedures.

## List of Abbreviations

POP: Plate Or Pin; ESIN: Elastic Stable Intramedullary Nailing; TEN: Titanium Elastic Nail; DMCF: Dislocated Midshaft Clavicular Fracture; ER: Emergency Room; DASH: Disabilities of Arm, Shoulder or Hand; DSMB: Data and Safety Monitoring Board.

## Competing interests

The authors declare that they have no competing interests.

## Authors' contributions

FJW provided the conception and design of the article, RMH is responsible for the design of the initial protocol. MGWD provided the statistical analysis. DHDL, SAGM, MHJV and EMMV were all responsible for analysis, writing and editing of the manuscript. All authors also gave final approval of the version to be published.
